# New light on ω-3 polyunsaturated fatty acids and diabetes debate: a population pharmacokinetic-pharmacodynamic modelling and intake threshold study

**DOI:** 10.1038/s41387-024-00262-w

**Published:** 2024-03-04

**Authors:** Ling Wang, Xiaomin Huang, Mingyao Sun, Tian Zheng, Luyan Zheng, Xiaolan Lin, Junshan Ruan, Fan Lin

**Affiliations:** 1 Shengli Clinical Medical College of Fujian Medical University, Fujian Provincial Hospital, Fuzhou, China; 2 Fujian Normal University Hospital, Fuzhou, China; 3 Department of Clinical Nutrition, Shengli Clinical Medical College of Fujian Medical University, Fujian Provincial Hospital, Fuzhou, China

**Keywords:** Type 2 diabetes, Fatty acids, Risk factors, Type 2 diabetes, Nutrition

## Abstract

**Objective:**

ω-3 polyunsaturated fatty acids (PUFA) are a key modifiable factor in the intervention of type 2 diabetes, yet recommendations for dietary consumption of ω-3 PUFA in type 2 diabetes remain ambiguous and controversial. Here, we revisit the subject in the light of population pharmacokinetic-pharmacodynamic (PPK-PD) modeling and propose a threshold for intake.

**Research design and methods:**

Plasma levels of ω-3 PUFA and glycosylated hemoglobin (HbA_1c_) were measured as pharmacokinetic and pharmacodynamic indicator, respectively. The nonlinear mixed effect analysis was used to construct a PPK-PD model for ω-3 PUFA and to quantify the effects of FADS gene polymorphism, age, liver and kidney function, and other covariables.

**Results:**

Data from 161 patients with type 2 diabetes in the community were modeled in a two-compartment model with primary elimination, and HDL was a statistically significant covariate. The simulation results showed that HbA_1c_ showed a dose-dependent decrease of ω-3 PUFA plasma level. A daily intake of ω-3 PUFA at 0.4 g was sufficient to achieve an HbA_1c_ level of 7% in more than 95% of patients.

**Conclusions:**

PPK/PD modeling was proposed as a multilevel analytical framework to quantitatively investigate finer aspects of the complex relationship between ω-3 PUFA and type 2 diabetes on genetic and non-genetic influence factors. The results support a beneficial role for ω-3 PUFA in type 2 diabetes and suggested the intake threshold. This new approach may provide insights into the interaction of the two and an understanding of the context in which changes occur.

Type 2 diabetes is a common endocrine disease, and its global prevalence is increasing yearly. Between 1990 and 2019, age-standardized disability-adjusted life-years due to type 2 diabetes increased by 27.4% (22.0–32.5). The burden for type 2 diabetes across countries increased with higher type 2 diabetes prevalence, and the main risk factors for the burden were high BMI, with a population attributable fraction of 63.2% and dietary risks, with a PAF of 27.5% [1]. Health care costs associated with type 2 diabetes are increasing, and it is estimated that by 2030, more than $2.2 trillion will be spent on type 2 diabetes [2]. Dietary and nutritional approaches for the prevention and management of type 2 diabetes are economical and well-accepted, in which immunonutrients are valued [3].

ω-3 PUFA, as an important immune nutrient, has pharmacological effects such as regulating blood lipid, reducing inflammatory response, and enhancing immunity. However, the role of ω-3 PUFA in type 2 diabetes remains a controversial topic. ω-3 PUFA is found to have beneficial effects on insulin sensitivity. ω-3 PUFA binds to the G protein-coupled receptor GPR120, leading to a decrease in cytokine production by inflammatory macrophages and improving signaling in adipocytes, thereby reducing insulin resistance in patients with type 2 diabetes [4]. Supplementation with ω-3 PUFA also has a protective effect against many complications of type 2 diabetes [2], such as ameliorating chronic neuroinflammatory diseases of the peripheral and central nervous system [5], normalizing catabolic disorders [6] and preventing muscle atrophy [6]. Conversely, it has been reported that the supplementation ofω-3 PUFA was either positively or insignificantly associated with type 2 diabetes [7, 8]. The ASCEND study found that among approximately 15,000 patients with type 2 diabetes, those taking 1 g of ω-3 PUFA supplements daily, compared with those taking 1 g of olive oil, did not show significant differences in cardiovascular disease incidence and all-cause mortality during a 7.4-year follow-up period [9]. Some other evidence from clinical trials and cohort studies likewise suggests that consumption of ω-3 PUFA is not beneficial or harmful for type 2 diabetes, whether supplementing with ω-3 PUFA -rich products or foods [10, 11].

Genetic and non-genetic factors make the relationship between ω-3 PUFA intake and type 2 diabetes more complicated. Fatty acid desaturases (FADS) gene polymorphisms have been reported to affect the role of ω-3 PUFA in type 2 diabetes. The endogenous metabolic process of ω-3 PUFA is mediated by delta-5-desaturase (D5D) and delta-6-desaturase (D6D), which are encoded by the genes of fatty acid desaturase 1 (FADS1) and fatty acid desaturase 2 (FADS2), respectively. Genetic variation in FADS genes is associated with reduced expression and activity of D5D and D6D, which affects the concentration ofω-3 PUFA [12]. While the potential role of FADS gene polymorphisms is plausible, further clinical studies are needed to better understand the extent of this role and the intake of ω-3 PUFA that would therefore need to be altered. In addition, the levels of nutrients in the body are influenced by gender, other drugs, etc. After supplementation with esterified forms of ω-3 PUFA, women had 2.7-fold higher plasma concentrations during the absorption phase (*p* = 0.0111), 1.8-fold higher plasma concentrations at 24 h (*p* = 0.0006) and 1.7-fold higher maximum concentrations (*p* = 0.0295) compared to men [13]. Statins have the ability to alter the concentrations ofω-3 PUFA. Statins have differential effects on the activities of the Δ6- and Δ5-desaturase enzymes, and studies indicate increases in activity (simvastatin, rosuvastatin, and pitavastatin) or decreases (atorvastatin) [14].

Although the positive and negative impact of ω-3 PUFA on type 2 diabetes have been investigated, and some of the influencing factors were also considered, many of these prior trials are limited by a lack of major dietary pattern shifts and memory errors of self-reported dietary. Quite a few studies were conducted years ago, creating potentially low generalizability to contemporary diets and clinical settings. The universal problems with all self-reported dietary assessment methods include underreporting, literacy level, reliance on memory, imprecise estimation of portion sizes, possible bias because of direct food intake, and lack of dietary details [15]. Compared with self-reported dietary consumption, circulating ω-3 PUFA level is not subject to recall bias and allows for objective assessment of individual ω-3 PUFA. In addition, ω-3 PUFA plasma level represents the combined influence of diet and metabolism and thus may better reflect bioavailable ω-3 PUFA intake. Yet, the relations between plasma levels of ω-3 PUFA and type 2 diabetes have been evaluated in relatively few studies, with wide variation and uncertain impact extent in study designs, outcomes, covariates, and statistical methodology [16]. Thus, for many scientists and clinicians, the role of ω-3 PUFA in type 2 diabetes, influenced by both genetic and non-genetic factors, needs to be unambiguously quantified by more reliable methods.

Interestingly, PPK/PD modeling opens a more precise way to explore the complex association between ω-3 PUFA and type 2 diabetes. PPK-PD allows precise analysis of the quantitative relationship between the analyte plasma concentration and the primary efficacy index in patients by establishing a mathematical formula, which includes the quantitative impact of demographic, clinical information, and other covariates [17]. PPK-PD has been successfully applied to drugs such as acyclovir [18], vedolizumab [19], Oxycodone [20], teicoplanin [21], providing valuable information for rational clinical use. However, there is still no literature on PPK-PD of nutritional elements, including ω-3 PUFA, which is a major deficiency in precision nutrition. PPK-PD is necessary to achieve the adjustment of ω-3 PUFA intake according to individual covariate differences, resulting in the design of individualized dietary or nutritional supplementation regimens to further improve efficacy and reduce side effects.

Herein, on the basis of ω-3 PUFA plasma level, we establish and validate a PPK-PD model of ω-3 PUFA in type 2 diabetes with the quantitative impact of genetic and non-genetic factors. Furthermore, based on this model, the intake regimen of ω-3 PUFA was optimized and proposed for patients with type 2 diabetes.

## Research design and methods

### Study design and participants

This was a two-center prospective clinical study, which was approved by the Ethical Committee of Fujian Provincial Hospital (Fujian, China) and registered in the Chinese Clinical Trial Registry (ChiCTR2000036210). Participants were selected from patients with type 2 diabetes in the East Street community and South Street community in Fuzhou, China, during 2020-2021. They were recruited and studied after obtaining written informed consent. Inclusion criteria included type 2 diabetes, 18 years of age or older, and regular diet. Exclusion criteria included incomplete clinical information, and conditions judged by the investigators to be inappropriate for this trial. Written informed consent was collected from all participants.

### Acquisition of covariables

Weight, height, and waist circumference were measured by trained professionals to standardized protocols, and body mass index (BMI) was calculated as weight divided by squared height (kg/m^2^). Lifestyle questionnaires were used to assess demographics, smoking status, medical history, and education level. The body composition was carried out by the multi-frequency bioelectrical impedance analysis on Inbody S10 (Biospace, Korea). HbA_1c_ was measured immunoturbidimetrically (Roche Cobas 6000 c 501, Mannheim, Germany). The serum biochemical parameters were determined using an AU480 Chemistry Analyzer (Beckman Coulter, USA). A routine blood test was performed on a Hematology Analyzer BC-5390 (Mindray, China).

### Measurement of ω-3 PUFA plasma concentration

Subjects were instructed to abstain from smoking, drugs, and eating for 12 h, prior to giving the sample. The plasma level of ω-3 PUFA was measured by Human ω-3 PUFA ELISA KIT (Jianlai Bio, Cat. #JL49574, China).

### Isolation, quantification of DNA, and determination of SNPs

DNA was extracted from whole blood using an animal DNA extract kit (TSINGKE, China) The products were sequenced using the Sanger sequencing method at Tsingke Biological Technology (Hangzhou, China).

### Analysis of dietary composition

Using the Food Frequency Questionnaire (FFQ), a trained dietitian asked participants about the average consumption frequency of each food during the previous year. The average daily intake of each nutrient was calculated by dietary analysis software (version 2.2.5.402, Wincome Corporation, Shanghai, China) [22].

### Analysis of population pharmacokinetics

A nonlinear mixed-effects model (NONMEM) was developed using the first-order conditional estimation-extended least-squares (FOCE ELS) method. One- or two-compartment models with first-order oral absorption and linear or nonlinear (Michaelis–Menten) elimination after oral administration were investigated to determine the optimal structural model. The covariates were examined one by one by forward inclusion and backward elimination methods. The −2 times logarithmic maximum likelihood value (−2LL) was defined as the objective function value (OFV) in the software fitting process, and the change in OFV was compared. If the difference in OFV after introducing a covariate (△OFV) >3.84, it means that the factor has a statistically significant effect on the model (*P* < 0.05). In this way, individually existing and meaningful covariates were screened out and a preliminary covariate model was established.

To further test the necessity of covariates meeting the above criteria in the final model of ω-3 PUFA, a more stringent statistical criterion was used (△OFV >6.64, *P* < 0.01) to diagnose the above initially constructed model of ω-3 PUFA covariates by backward elimination. If the △OFV of the model after excluding a covariate was >6.64, the factor had a statistical effect on the target model (△OFV >6.64, *P* < 0.01) and was retained in the model. Conversely, it was excluded. The final covariate model for ω-3 PUFA, i.e., the final model for ω-3 PUFA, was established.

### PPK/PD analysis

After the final PPK model was established, the Bayesian Maximum Posterior Probability Method was used to estimate the individual pharmacokinetic parameters, which were introduced into the data file for pharmacodynamic modeling. The modeling process of the pharmacodynamic model is the same as that of the pharmacokinetic model.

### Model evaluation

Goodness-of-fit plots and bootstrap were used to evaluate the accuracy and stability of the model.

### Model-based simulations

To recommend the intake of ω-3 PUFA for different HbA_1c_ levels, various dosing simulations based on the established final model were performed. The parameter estimates, as well as their interindividual variability, were used to simulate the plasma level with 1000 replicates.

### Application of the final model

The final model was applied to 49 patients with type 2 diabetes and the predicted values were compared with the true values. The median prediction error (MDPE, Eq. 1), median absolute prediction error (MAPE, Eq. 2), and relative prediction error (PE%, Eq. 3) within ±20% (F_20_) and PE% within ±30% (F_30_) were calculated to assess the accuracy and precision of the model. The predictive performance of a candidate model was considered satisfactory if the investigated models met all the standards (MDPE ≤±20%, MAPE ≤30%, F_20_ ≥ 35%, and F_30_ ≥ 50%).

1 $${\rm{MDPE}} \% =\frac{1}{N}\sum {PE}\times 100 \%$$

2 $${\rm{MAPE}} \% =\frac{1}{N}\sum {\rm{|}}{PE}{\rm{|}}\times 100 \%$$

3 $${\rm{PE}} \% =\left(\frac{{{PRED}}_{i}-{{OBS}}_{i}}{{{OBS}}_{i}}\right)\times 100 \%$$

PRED_*i*_ is the prediction value of the ith patient, and *OBS*_*i*_ is the observed value of the ith patient.

### Statistical analysis

The Kruskal–Wallis test were applied to check the differences in ω-3 PUFA plasma concentrations among the FADS phenotype groups of rs174545, rs2072114, rs174602, rs174616, and rs174547. *P* < 0.05 was considered statistically significant. Statistical analysis was performed using GraphPad Prism version 8.0.1 (Dotmatics, Boston, Massachusetts, USA).

### Statistical Software

Phoenix NLME program (version 1.2, Pharsight Corporation, USA) and R software (version 3.3.1, http://www.r-project.org) were used.

## Results

### Demographic characteristics

The demographic and physiological characteristics of patients are summarized in Table 1.

**Table 1:** Demographic and clinical data.

Characteristic	Mean ± SD	(Median, range)
Age (years)	67.16 ± 7.29	(67,43–85)
Sex (male/female) (n)	64/97	-
Height (cm)	159.91 ± 8.68	(159,136–184)
Weight (kg)	63.52 ± 10.93	(63,39–102)
BMI (kg·m^−2^)	24.82 ± 3.77	(24.61,16.02–43.79)
Body Fat Mass (kg)	19.19 ± 5.72	(18.8,8.0–41.9)
Waistline (cm)	82.1 ± 8.39	(81.3,62.1–111.3)
Minerals (kg)	2.37 ± 0.44	(2.3,1.49–4.34)
Visceral fat area (cm^2^)	93.51 ± 60.23	(89.5,32.4–737.1)
Basal Matabolic Rate (kcal)	1313.14 ± 186.5	(1270,978–2073)
WBC (×10^9^ /L)	5.95 ± 1.36	(6.09,2.18–10.19)
PLT (×10^9^ /L)	228.34 ± 40.97	(226,103–335)
HG (g/L)	139.25 ± 15.2	(138,109–178)
ALT (U/L)	28.52 ± 13.93	(25.4,1.12–76.55)
AST (U/L)	25.43 ± 11.77	(22,1.64–103.00)
TBIL (μmol/L)	13.21 ± 6.62	(12.1,3.77–55.20)
SCr (μmol/L)	64.89 ± 19.34	(61.8,28.24–128.71)
BUN (mmol/L)	4.90 ± 1.43	(4.87,2.20–11.03)
UA (μmol/L)	414.74 ± 114.47	(401.3,209.8–813.7)
eGFR (ml/min)	85.63 ± 29.06	(81.4,27.50–195.43)
TG (mmol/L)	1.89 ± 1.33	(1.57,0.66–10.50)
TC (mmol/L)	4.96 ± 1.19	(4.8,1.25–8.01)
LDL (mmol/L)	2.78 ± 0.93	(2.66,0.74–5.17)
HDL (mmol/L)	1.38 ± 0.38	(1.28,0.77–2.49)
Hcy (μmol/L)	16.48 ± 6.08	(15.4,7.1–45.5)
TP (g/L)	43.91 ± 2.47	(43.7,36.9–52.9)
ALB (g/L)	29.56 ± 3.55	(29.3,19.4–38.7)
FPG (mmol/L)	6.74 ± 2.39	(6.3,3.6–22.5)
HbA_1c_		
%	6.31 ± 1.91	(5.7,2–14.1)
mmol/mol	49.4 ± 20.6	(42.9,3–133.4)
SNPs		
rs174545(CG/GG/CC)(n)	92/45/24	-
rs2072114(AG/AA/GG)(n)	46/99/16	-
rs174602(CT/TT/CC)(n)	82/68/11	-
rs174616(AG/GG/AA)(n)	84/73/4	-
rs174547(CT/CC/TT)(n)	97/43/21	-
hypertensive medicine (Y/N) (n)		
ACEI/ARB	17/144	-
β- receptor blocker	5/156	-
Calcium channel blocker	21/140	-
Diuretics	4/157	-
Hypoglycemic drugs (Y/N) (n)		
Biguanide	45/116	-
α- glycosidase inhibitors	35/126	-
Insulin sensitizer	4/157	-
DPP-4 inhibitors	5/156	-
GLP-1 receptor agonist	0/161	-
Insulin secretagogue	48/113	-
SGLT-2 inhibitors	3/158	-
Insulin	13/148	-
Lipid-lowering drugs (Y/N) (n)		
Statins	10/151	-
Niacin	0/161	-
Cholesterol absorption inhibitors	1/160	-
ω-3 PUFA plasma level(g/L)	0.0319 ± 0.0091	(0.0299,0.0107–0.0508)
ω-3 PUFA intake (g)	1.14 ± 1.19	(0.774,0.036–6.426)

*WBC* white blood cell, *PLT* platelets, *HG* hemoglobin, *ALT* alanine aminotransferase, *AST* aspartate aminotransferase; *TBIL* total bilirubin, *SCr* serum creatinine, *BUN* blood urea nitrogen, *UA* uric acid, *eGFR* estimated glomerular filtration rate, *TG* triglyceride, *TC* total cholesterol, *LDL* low density lipoprotein, *HDL* high-density lipoproteins, *Hcy* homocysteine, *ALB* albumin, *HbA*_*1c*_ glycosylated hemoglobin, *SNP* single nucleotide polymorphism, *ACEI* angiotensin converting enzyme inhibitors, *ARB* angiotensin receptor blocker; *DPP-4* dipeptidyl peptidase-4, *GLP-1* glucagon-like peptide-1, *SGLT-2* sodium-dependent glucose transporters-2.

### PPK analysis

The two-compartment model with first-order elimination was used as the base model in this study, proved that the basic model of ω-3 PUFA fitted well. In the covariate screening, the results showed that high-density lipoprotein (HDL) had significant effects on the pharmacokinetics of ω-3 PUFA (*P* < 0.05). The covariates were substituted to obtain the final model. The equations of the final model are as follows:

$${\rm{Ka}}=1.175* {({\rm{HDL}}/1.38)}^{0.007* \exp ({\rm{\eta }}{\rm{Ka}})}$$

$${\rm{V}}=26.151* {({\rm{HDL}}/1.38)}^{(-0.535)* \exp ({\rm{\eta }}{\rm{V}})}$$

$${\rm{CL}}=0.411* {({\rm{HDL}}/1.38)}^{0.285* \exp ({\rm{\eta }}{\rm{CL}})}$$

Ka is the absorption rate constant, V is the volume of distribution, CL is clearance, and η is a random variable distributed.

**Fig. 1:**
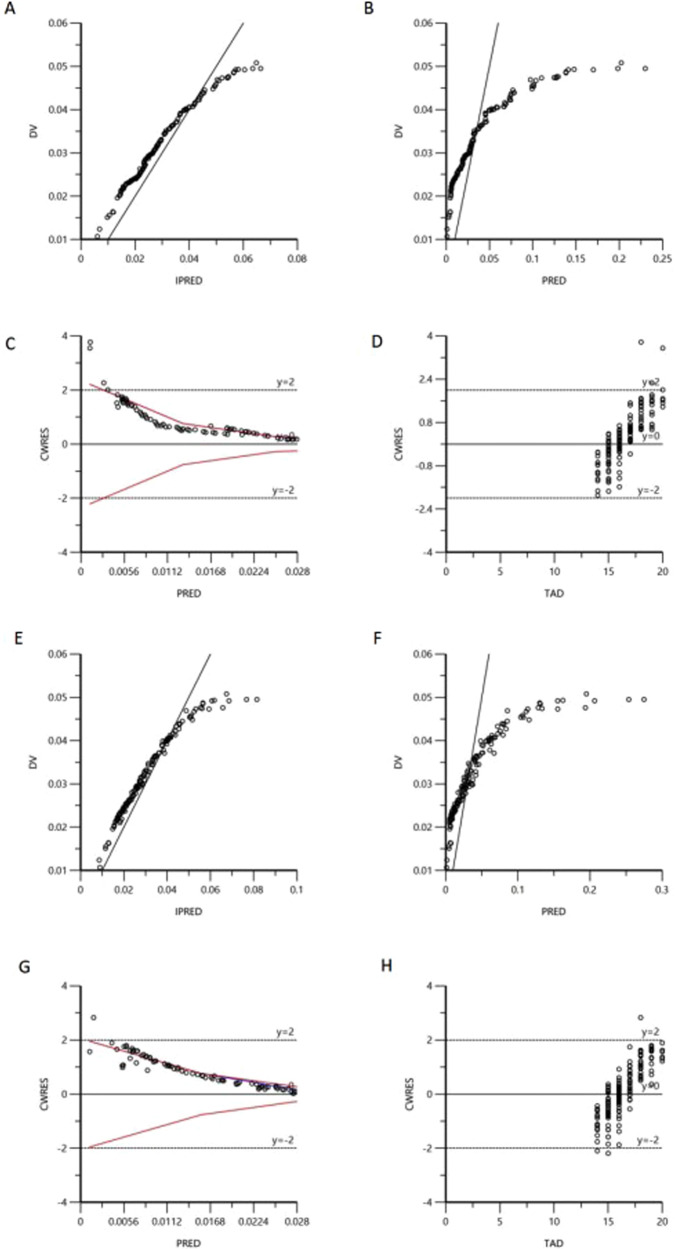
The diagnostic plots for the basic/final PPK model. **A** Scatter plot of individual predicted vs. observed values in basic model; **B** scatter plot of population predicted vs. observed values in basic model; **C** scatter plot of conditional weight residual vs. population predicted value in basic model; **D** scatter plot of conditional weight residual vs. time after dose (TAD) in basic model; **E** scatter plot of individual predicted vs. observed values in final model; **F** scatter plot of population predicted vs. observed values in final model; **G** scatter plot of conditional weight residual vs. population predicted value in final model; **H** scatter plot of conditional weight residual vs. time after dose (TAD) in final model. In the scatter plot, circle indicates blood concentration of ω-3 PUFA, black line indicates accurate line, red line indicates trend line.

The OFV decreased by 9.15 in the final model compared with the base model, indicating that the incorporated covariates contributed to substantial model improvement. The PRED strongly deviated from the observed concentrations in the base model, but the PRED agreed with the detected values (DV) in the final model in the scatter plots of DV vs. PRED (Fig. 1A, E). In addition, CWRES in the final model were more uniformly distributed within the accepted range (y = ±2). By contrast, the two average CWRES trend lines of the base model slightly extended outward at the end (Fig. 1C, G). In brief, the final model was significantly improved in terms of the GOF and allowed more accurate prediction of ω-3 PUFA levels.

In the bootstrap for the final model, all the 1000 replications ran successfully. The population parameter estimates were close to the median values from bootstrapping analysis and fell within 95% CIs (Table 2), suggesting that the final model was robust and accurate.

**Table 2:** Population mean estimates of the final model parameters and bootstrap results.

	Final model (CV%)	Bootstrap results	
		Median	2.5th–97.5th percentile
Ka	1.175 (0.096)	1.184	0.005–6.346
V (L)	26.151 (1.451)	25.99	12.995–204.661
CL (L·h^-1^)	0.411 (0.000)	0.568	0.37–92.616
σ	0.354 (0.000)	0.380	0.008–0.49
E0 (%)	5.641 (2.42)	5.58	5.528–6.021
IC50 (g/L)	0.090 (4.776)	0.089	0.089–0.098
Imax (%)	0.597 (8.262)	0.615	0.425–0.615
σ	0.354 (0.000)	1.615	0.354–0.354

### PPK-PD model

No covariates were found to have a significant effect on the pharmacodynamic parameter, HbA_1c_. Thus, the basic model is the final model, which can be used to derive the corresponding HbA_1c_ values from the plasma concentration of ω-3 PUFA and vice versa. The resulting model are as follows:

$${\rm{IC}}50=0.089* {{\rm{e}}}^{({\rm{\eta }}{\rm{IC}}50)}$$

$${\rm{E}}0=5.5796* {{\rm{e}}}^{({\rm{\eta }}{\rm{E}}0)}$$

$${\rm{I}}\max =0.615* {{\rm{e}}}^{({\rm{\eta }}{\rm{I}}\max )}$$

E0 is the baseline effect, IC50 is the drug concentration corresponding to the 50% maximum inhibitory effect, and Imax is the maximum inhibitory effect.

The conditional weight residuals of PPK/PD prediction values are within ±3 standard deviations. The high agreement between the individual predicted values of the model and the measured values indicated that the prediction accuracy of the model was good. The 95% CI of the bootstrap parameters included the final model parameters, suggesting that the model was stable.

### Dose simulation of ω-3 PUFA intake to meet the control target of HbA_1c_

**Fig. 2:**
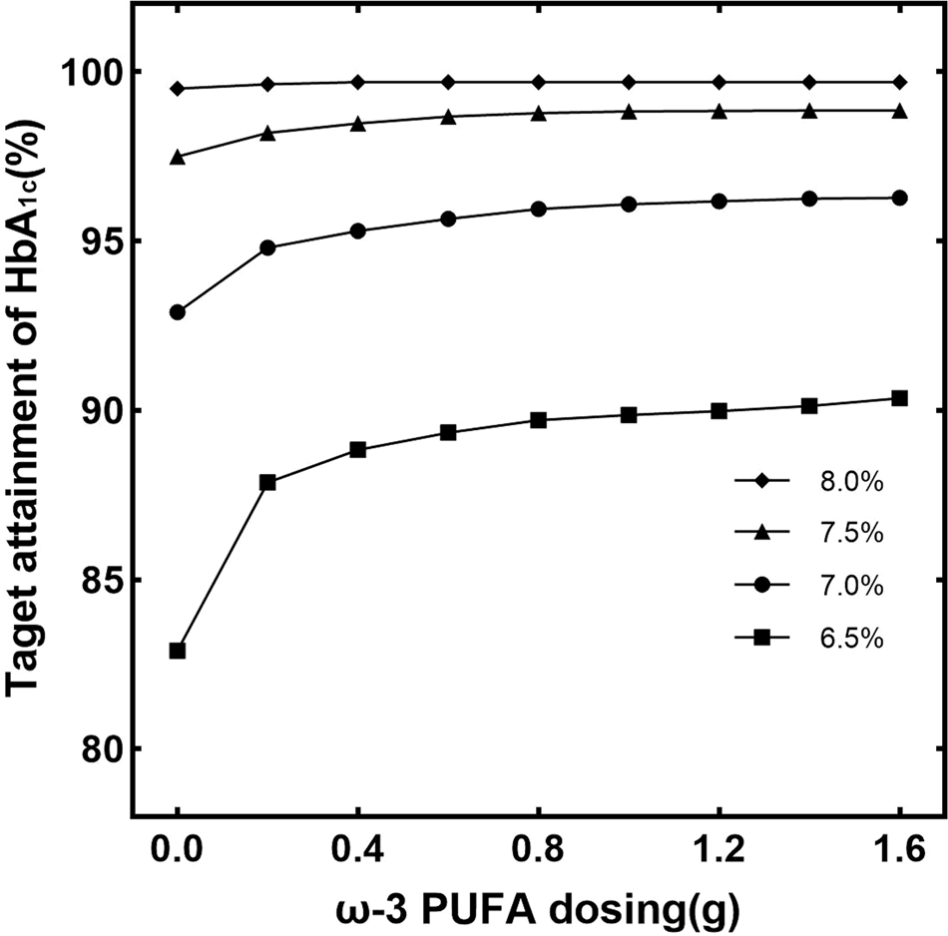
Probability of achieving HbA_1c_ target ω-3 PUFA with 0.0–1.6 g/d oral dosing regimens in different HbA_1c_ target groups.

The probability of attaining different target levels of HbA_1c_ with various dosing regimens of ω-3 PUFA is shown in Fig. 2. A daily intake of ω-3 PUFA at 0.4 g was sufficient to achieve HbA_1c_ level of 7% (56.9 mmol/mol) in more than 95% of patients. However, if the control target for HbA_1c_ is 6.5% (51.5 mmol/mol), a daily intake of 1.3 g ω-3 PUFA would result in 90.08% of patients reaching the target. As can be seen, patients requiring strict control of HbA_1c_ are expected to have a higher intake of ω-3 PUFA.

### Application of the model

In 49 type 2 diabetes cases, the final PPK model had MDPE 11.23%, MAPE 29.00%, F_20_ 38.78%, and F_30_ 81.63%, and the final PPK-PD model had MDPE −6.87%, MAPE 15.32%, F_20_ 69.39%, and F_30_ 85.71%. Prediction-based diagnostics showed that both models met all the standards (MDPE ≤±20%, MAPE ≤30%, F_20_ ≥ 35%, and F_30_ ≥ 50%), indicating satisfactory predictability.

### The differences between groups of ω-3 PUFA plasma concentrations of different genotypes

**Fig. 3:**
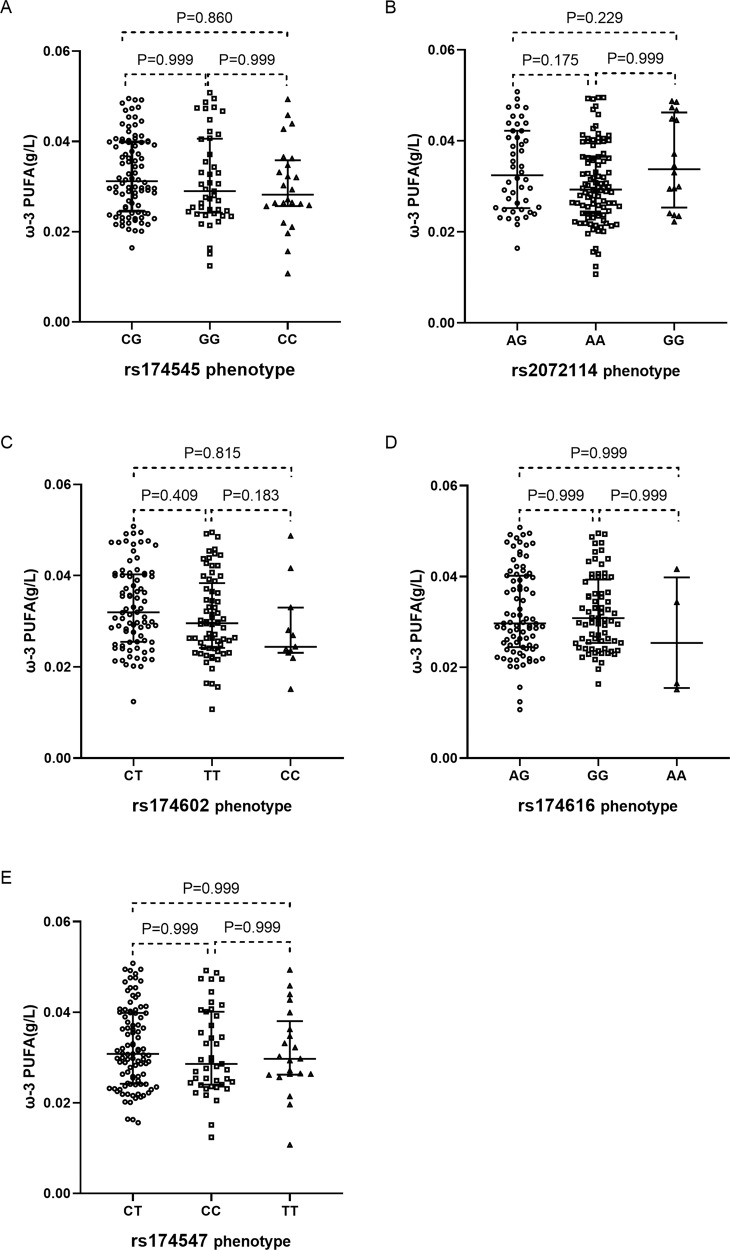
Distribution of ω-3 PUFA plasma concentrations among the five phenotype groups. Distribution of ω-3 PUFA plasma concentrations among rs174545 (**A**), rs2072114 (**B**), rs174602 (**C**), rs174616 (**D**) and rs174547 (**E**) phenotype groups. Kruskal–Wallis test was made for pairwise comparisons. Data are expressed as the median ± interquartile range.

There was no significant difference in ω-3 PUFA plasma concentrations among the FADS phenotype groups of rs174545, rs2072114, rs174602, rs174616, and rs174547 (Fig. 3).

## Conclusions

To our knowledge, this study is the first to establish a PPK/PD model for ω-3 PUFA in patients with type 2 diabetes, which enables the “point-to-point” prediction of HbA_1c_ and ω-3 PUFA levels. This study has some strengths different from related investigations. First, rather than simply analyzing the correlation between ω-3 PUFA and type 2 diabetes, the relationship between ω-3 PUFA levels and HbA_1c_ was quantified, which is a mathematical model for individual calculations, providing specific references for the development of individualized supplementation programs. Second, the impact of individual differences and confounding factors was fully considered. The variability of concentration-effect influenced by demographic information, genetic factors, drugs and other factors in patients was quantitatively examined. Finally, combined with clinical feedback data from each patient after taking the reference intake, simulations and predictions can be performed again or repeatedly to achieve continuous improvement in the accuracy of individual prediction.

Our study indicates that the pharmacodynamic effect of ω-3 PUFA in type 2 diabetes depends on the amount ingested. Given sufficiently higher doses, the HbA_1c_-lowering effect of ω-3 PUFA is enhanced. A systematic review and meta-analysis, including 41 prospective cohort studies with a total of 1,197,564 participants also showed dose-dependent effects of ALA. In the dose-response analysis, a 1 g/day increase in ALA intake (equivalent to one tablespoon of canola oil or 0.5 ounces of walnut) was associated with a 5% lower risk of all-cause (0.95, 0.91–0.99, I2 = 76.2%, *n* = 12) and CVD mortality (0.95, 0.91–0.98, I2 = 30.7%, *n* = 14). The pooled relative risks for the highest compared with lowest tissue levels of ALA indicated a significant inverse association with all-cause mortality (0.95, 0.90–0.99, I2 = 8.2%, *n* = 26). Also, based on the dose-response analysis, each 1 standard deviation increment in blood concentrations of ALA was associated with a lower risk of CHD mortality (0.92, 0.86–0.98, I2 = 37.1%, *n* = 14) [23]. As for pharmacokinetics, the present study yielded the population Ka, V, CL, and other parameters of ω-3 PUFA, which have not been seen in the literature. From these parameters, it can be found that ω-3 PUFA is completely absorbed in less than 1 h after ingestion, and are mainly distributed in plasma and other body fluids after entering the body, with a slower rate of clearance out of the body [24, 25]. The half-life of ω-3 PUFA was deduced from the pharmacokinetic formula to be ~43.86 h [24–26], which is consistent with the half-life of total EPA and DHA reported by Hitoshi Shimada et al. [27]. As can be seen, ω-3 PUFA is highly bioavailable and are maintained in vivo for a long time.

There is no consensus on the recommended daily dietary intake of ω-3 PUFA supplementation for patients with type 2 diabetes, with a large recommended dose range of 1g–5.9 g/day [28, 29]. Such a wide range of recommended supplementary dose has brought great confusion to patients with type 2 diabetes and their doctors. Related studies simply examined the correlation between the intake of ω-3 PUFA and HbA_1c_, in which many were not derived from real ω-3 PUFA levels in vivo, and some did not even fully check the effects of genes and other factors. Moreover, the algorithms of these studies were inferior in accuracy to the population pharmacokinetic algorithm and apparently cannot obtain pharmacokinetic parameters and individual variations. This study provides a more precise model, the PPK-PD model between ω-3 PUFA and HbA_1c_, which not only indicates the intake thresholds of ω-3 PUFA required for different HbA_1c_ targets in the general diabetic population, but also allows the calculation of individual doses for each patient in different situations. 0.4 g of ω-3 PUFA per day can keep HbA_1c_ at 7% (56.9 mmol/mol) in more than 95% of patients with type 2 diabetes, yet the global average dietary intake of ω-3 PUFA is 0.10 g/day [30]. Therefore, patients with type 2 diabetes need additional health products or medications of ω-3 PUFA. Such a supplement is also reassuringly safe. No serious adverse events occurred after taking ω-3 PUFA supplement up to 3 g/d in small trials [31, 32]. A systematic review of ω-3 fatty acids on the primary and secondary prevention of cardiovascular diseases found that increased intake of ω-3 PUFA did not raise the risk of bleeding [33]. A detailed secondary analysis of the OPERA trial showed that the intake of high-dose ω-3 PUFA (preoperative loading dose of 8–10 g for 2–5 days and postoperative dose of 2 g/d) did not increase the risk of bleeding in 1516 patients undergoing cardiac surgery recruited from multiple countries [34]. A meta-analysis of several large prospective observational studies found that consumption of fish rich in ω-3 PUFA did not have a significant impact on the risk of most cancers [35], and the same results were found in randomized trials of fish oil [36].

This study found that the effect of ω-3 PUFA on HbA_1c_ was influenced by HDL. In patients with lower HDL, the clearance of ω-3 PUFA was accelerated, and the efficacy of reducing HbA_1c_ was correspondingly diminished. This may be an explanation for the inflection point of the HDL level in improving HbA_1c_. A cross-sectional study observed a U-shaped association between HDL-C and HbA_1c_ in patients with type 2 diabetes aged 60 years and older, with an HDL-C inflection point of 60 mg/dL [37]. The mechanism of the correlation between HDL and the pharmacokinetics of ω-3 PUFA can be explained by some of the current literature reports. Protein-based subspecies of HDL, especially those containing apolipoprotein E (apoE) or apolipoprotein C3 (apoC3), offer a glimpse of a vast metabolic system related to atherogenicity, obesity, type 2 diabetes, and other diseases. ApoE stimulates several processes that define reverse cholesterol transport through HDL, specifically secretion of active HDL subspecies, cholesterol efflux to HDL from macrophages involved in atherogenesis, size enlargement of HDL with cholesterol ester, and rapid clearance from the circulation. Dietary unsaturated fat, including ω-3 PUFA, stimulates the flux of HDL that contains apoE through these protective pathways. Effective reverse cholesterol transport may prevent related diseases. In contrast, apoC3 abrogates the benefit of apoE on reverse cholesterol transport, which may account for the association of HDL that contains apoC3 with type 2 diabetes and other diseases [38]. The consumption of ω-3 PUFA, particularly EPA and DHA, contributed to a greater decreasing tendency in plasma insulin, HbA_1c_, TC, TG, and BMI measures. The longer the duration of the intervention, the more effective the ω-3 PUFA appeared to be on glycemic control and lipid levels [39].

α-linolenic acid in vegetable oils such as perilla oil, flaxseed oil, and chia seed oil can be converted to ω-3 PUFA by fatty acid desaturases. This transformation rate was influenced by polymorphisms of the gene FADS, which encodes fatty acid desaturase. However, the effect of FADS polymorphism on ω-3 PUFA plasma level was not found in this study. One reason is that the metabolic ratio of ω-3 PUFA mediated by D5D and D6D is very low, generally less than 10% [40]. Another possible reason is that this study examined multiple factors, and the FADS gene polymorphism had a significantly smaller effect compared to other factors.

The limitation of this study is that it is a single-center investigation that has not been validated by other centers. Variations of living conditions and dietary habits in different regions may lead to deviations. In addition, the association between high doses of ω-3 PUFA and the risk of adverse events needs to be observed in a larger population. Therefore, multi-center studies with larger sample sizes and longer periods are still expected for further validation to provide more scientific evidence.

In conclusion, the relationship between ω-3 PUFA and HbA_1c_ was quantitatively described in patients with type 2 diabetes in the form of PPK-PD modeling, and HDL was found to be a significant influencing factor. The validation showed that the final model was stable with a good fit. ω-3 PUFA intake of 0.4 g/d can achieve an HbA_1c_ level of 7% (56.9 mmol/mol) in more than 95% of patients with type 2 diabetes, and higher doses may be required for more stringent HbA_1c_ targets. In addition, the application indicated that the model had good validity and predictiveness. Our findings not only shed new light on the interrelationship between ω-3 PUFA and type 2 diabetes, but also reveal new insights into the variability involved. PPK-PD modeling is an optional tool for exploring the fine diabetes-nutrient relationships.

## Data Availability

The datasets generated during and analyzed during the current study are available from the corresponding author on reasonable request.
